# Supplementary material for: Lineal Discrimination of Horses and Mules. A Sympatric Case from Arauca, Colombia
Source: Animals (Basel). 2020 Apr 13;10(4):679. doi: 10.3390/ani10040679 (PMC7222724; doi:10.3390/ani10040679)
Supplement: Supplementary file 1 [file animals-10-00679-s001.pdf]

Supplementary files: Lineal discrimination of Horses and Mules. A sympatric case from Arauca, Colombia

Arcesio Salamanca-Carreño, Jordi Jordana, Rene Alejandro Crosby-Granados, Jannet Bentez-Molano and Pere M. Parés-Casanova

Table S1. Crude values for analysed traits. Values expressed in cm.

Species	Sex	Age	LCO	ALC	ALGR	ANC	PT	DDE	DB	AL	ANESP	ALES	ANT	PCA	LCÑ	LCA	ACA	LCR	ACR	LC	AC	LORE	LGR	AGR	ACO	ORE	Finca
cb	M	7	125.3	131	136	112.8	155	57	42	125	26.3	66	20.2	17.5	18	52	20.5	17	12.5	35	16.5	15	45	46	46	8.5	Zamuracos
cb	M	6	131	137.2	136.2	115.2	155	59.3	42.3	130	31.5	72.8	34.5	24	19	55	17.5	19.2	17	35.8	19.7	16.5	46.7	43.9	53.7	8	El_Secreto
cb	M	5	132.5	136.5	134.6	119	150	55.2	41	129.5	29.5	72.5	31	21.5	20	54.5	20.2	16.1	16	38.4	13.3	16	44.3	43.5	49.7	10	El_Secreto
cb	M	2	123.7	122.7	126	110.6	152	55.6	42.6	120.2	29	65.7	30.5	22.5	19	54.1	20	17	12	37.1	15.3	14	42.7	39.4	45.6	7	Belencito
cb	M	8	120	131	131	114.3	155	56	43	123.6	26.2	68.5	19	16	17	51.5	21.3	15.5	13	36	17	16	45	43	49.8	7.5	Zamuracos
cb	M	9	137	136	136	127	157	59	35	129	29	67.5	23	18	19.5	56	21	17	12	39	18	16	43	41	49	7.5	Bahía
cb	M	7	124	127.3	131.9	119.3	147	56	42	124	26.5	68	21.2	15.5	15	53	21	16	12	37	16	16	45	42	49.4	8.5	Zamuracos
cb	M	2.5	125.4	126	126	114	142	56	37	124	24	71	17.3	16	16	52.5	16.5	15	12	37.5	17	13	41	37.7	50	8	Zamuracos
cb	M	6	122.3	129.5	127.8	114.7	149	56	37	120.5	26	66	22	17.5	16.5	55	21	18	14	37	16.5	12	45	44	45.7	8	Zamuracos
cb	M	4	132	133	137.9	119	157	59.3	47.9	127	35	64.5	36	25.5	21	55.5	19.5	20	13.5	35.5	17	16	43.5	45	51	8	B._Aires
cb	M	5	127	138	141	126	154	58	40	134	24	74	19	17	16	54	21	16	12	38	17	15	44	44	49	7	Bahía
cb	M	4.6	137	136	141	128	156	56	49	124	32.2	66.5	34.5	26	21	55.5	18	19.5	12	36	16.8	16	43.5	44	56	9	B._Aires
cb	M	7	137	139	139.4	129.7	160	60	43.8	134	30.1	74	33.4	22.5	19.5	57	20	17	13	40	18	14	43.6	43.2	54	7	Belencito
cb	M	6	129.5	134	134	116	163	62	48.1	127	29.2	70	35	23	21	54.1	19	18	12	36.1	17.2	17	40	42.5	43.4	8	Belencito
cb	M	14	131	133	138	130	157	61	37	129	28	69	23	18.8	20	56	21	17	12	39	17	16	43	44	52	8.5	Bahía
cb	M	9	124	131.7	132.7	121.3	151	57	36	124.5	26	65	21	17.5	17	53	20	16	11	37	16	16.5	44	43	46	7	Bahía
cb	M	10	119.8	130.7	131	115.3	151	56	42	124.3	27	70	18.5	16	17	51	20	16	12	35	16	14.5	45	43	49.8	7.5	Zamuracos

cb	M	4	128.8	133	131	119.9	145	55	35	125	23	68	17.3	16	16.5	51.7	19	16	14	35.7	14.8	15.5	45	40	48.3	7.5	Victoria
cb	M	3.6	134.3	133	134	122	148	57.3	42.6	128	29.6	73	35.3	24	26.5	52.5	20.1	16	13.3	36.5	15.8	16	45	42	53	8.5	Belencito
cb	M	2	128.2	135	137	118	146	56	35	124	25	71	22	19	20	55	20	17	11	38	16	14	43	39	48	7	Bahía
cb	M	2.6	123.5	131	132.5	118	147	56	38.2	127.5	27.5	69.5	28.3	23.5	23	49.7	20.6	18.5	15.8	31.2	16.6	17	42	39	52	7.5	Mercedes
cb	M	6	129.5	135	136.5	122	152	58.3	36	131	33.2	71.5	34	20	18.5	60	24	24	24	36	27	15	43	43.5	50.3	8	Mercedes
cb	M	7	135	134.5	133	115.7	152	53.5	38.7	129.5	30.8	69.9	35	25	23	54	21.1	19	15	35	15.7	15.5	42.7	40.7	49.7	9	Mercedes
cb	M	4	126	135	140	129.7	150	58	32	124	27	68	23	18	19	54	21	16.5	13	37.5	18	14	43	42	50	8	Bahía
cb	M	8	128	129.3	134.8	115	156	58.7	36.6	125.5	36	65.3	32.7	25	18.5	49.7	25.8	17	18	32.7	15.5	18	43.6	40.7	43.3	8.5	Mercedes
cb	M	7	134.5	135.7	137.3	123.5	154	56.5	42.5	124.7	35	70	34	23	20.5	53	20.7	20.8	20.5	32.2	17	20	45.5	40.8	46.3	7.5	Mercedes
cb	M	9	133.8	130.5	134.5	117.7	152	57	38.5	126.3	30	73	31.8	24.5	19	49	20	19.5	18	29.5	18	17	46	40.1	48.4	8	Mercedes
cb	M	19	126.3	124.7	124.4	110.8	148	54.6	46	117.5	26.7	67	32.8	23.5	17	55	19.4	15	12	40	16.2	16.5	42.5	40.3	46.4	8	B._Aires
cb	M	5	136.3	140.7	140	125	171	67.5	47.8	134	32.5	73.5	37.8	21.5	23	56.5	20.5	15.6	17.2	40.9	16.8	17	39.2	42.9	51.1	8.7	Ranchito
cb	M	6	124.2	131	130	108.5	150	54.6	42	126	29.3	67	34	22.5	16.5	53.7	18.8	16.1	12.1	37.6	15.1	14.5	44	37	50.4	8.5	B._Aires
cb	M	14	128	139	142.2	129.2	164	59.7	45.6	132	35.3	80	39.6	19	18.8	58.2	21	16.3	16.2	41.9	18.7	15	47.7	43.6	53	8.5	Ranchito
cb	M	8	138.8	140.5	145.5	129	177	64.9	48.2	139	36	72.3	40	19	18.8	57.3	19.7	19.5	15.5	37.8	18.5	15.2	49	46.5	49.6	8.2	Ranchito
cb	M	7	128	136	135.8	120.5	161	61	40.9	131.5	32.2	71.5	33.4	20.5	18	55.2	20.1	17.6	16.2	37.6	17.3	14	43.6	40.8	50	9	El_Secreto
cb	M	6	123	128.3	132.8	116.4	146	56	36	124.7	27	68.5	18	16	17	53	19	16	12	37	15.5	15	45	41.5	48.5	8	Zamuracos
cb	M	4	124	135	137.5	123.5	149	57	44	130	28	72.5	25	17	17	54	21	14	13	40	16	15	42	38	63	8	Arenosa
cb	M	5	140.2	132	133.4	120	154	56.5	41.3	126.7	31.6	68	35	22	19	57.3	20.5	19.3	16.1	38	17	14.5	42.6	42.6	46.5	9	El_Secreto
cb	M	8	128	129.8	129.4	115.5	147	55	39	121	24.2	68	17	16	15.5	54	20	15	11	39	15	14	42	43	51.3	8	Zamuracos
cb	M	7	117.4	128.3	132.5	133.2	146	55	37	124.3	26	70	20	16.5	15	51	19	15	12	36	16	14	34	39	48.5	7	Delicias
cb	M	9	136	131.8	132	119	157	59	43	126	30	69	21	17	16	56	21.5	18.5	14	37.5	17.5	13.5	50	42	50	7.5	Zamuracos
cb	M	7	131	131	133	118	155	57	39	126	28	64	22	17	18	55	20	16	12	39	16	14	44	40	48	6.5	Bahía
cb	M	10	125	127.5	127	105.5	141	52	39.5	121	26.8	67.3	29.1	21	18	52	16.8	16	11.3	36	16.1	16.6	40.3	36	46	8.5	B._Aires
cb	M	4	127	128.5	132	119	139	52	29	123	25	70	19	16.5	17	54	21	16	12	38	17.5	16	43	39	50	7	Bahía
cb	M	2	121	119.3	123	113.4	137	51	28	115.4	20	64	18	16.5	14.3	52	19.3	15.5	12	36.5	15.5	15	44	37	47	7.5	Bahía
cb	M	6	138	140	140	124	158	57.3	39.5	133	30.1	79	24.2	18	19	55	21	14.5	13	40.5	17	14	44.7	43	54	8	Libertad
cb	M	2	130	133	133	119.5	156	57	42	127	30	69	24	17.5	19	54.4	21.1	17.3	14	37.1	17.2	14.5	45.3	40.5	44.5	8	Libertad

cb	M	3	138	144	144	124	162	62	43	137	31	74	25	17	18	58	21.2	15.2	13.7	42.8	17.5	16	44.2	46	48.5	7.5	Libertad
cb	M	5	125	135	132.5	120.8	153	56.5	41.5	130	29	71	23	17	18	53.7	20.1	13	14	40.7	17	15	44	39	53	8	Cabañas
cb	M	8	144	142	142.5	125	165	61	46	136.5	31	71	29	18.5	18	59	23	17	14.5	42	18.5	16	48	46.8	51	8.5	Cabañas
cb	M	3	140	134	133.2	119	156	58.1	38	127.5	29	70	23	18	17	58	22	17	13.3	41	17.5	16.5	47.6	40.4	56	8.5	Cabañas
cb	M	6	133.5	135	138	123	155	57	34.7	130.2	31.5	73	34.5	23	21	61.5	23.5	22.5	18.5	39	16.5	17	44.1	44.4	53.7	8.5	Mercedes
cb	M	11	126.5	138	134.2	118	161	59	45.8	131	32.1	73	34.8	24	19	49	18	15	10	34	15.7	15	46.1	42.6	42	8	B._Aires
cb	M	8	145	139	142	124	164	59.4	38	130	29	72	23.5	19	16	59	22	17	13	42	19.3	17	46.5	48.3	58	8.5	Cabañas
cb	M	4	135.5	127	128	117.5	155	57.3	42.5	122	29	64	21.4	16.5	17	56	22	15	14.5	41	17	14	44.5	42	45.2	7	Cabañas
cb	M	4	134	138	139	128	163	57.2	42.5	134	30	74	19	18	17	59.5	23	19.7	14.5	39.8	18	16.5	47	48	56	7	Cabañas
cb	M	5	140	137	142	128	162	60.5	43	136	30	68.5	21.7	19.5	18	56	21	16	13	40	18	16	46	43	56	7.5	Cabañas
cb	M	4	137	139	135.5	114	161	57	47	131	27	70	21	17	17	57.5	21	15	13	42.5	18	15	46	44	53.5	8	Cabañas
cb	M	3	136	135	132	120.3	155	56.5	42	130	28.5	70.5	23.5	17	17	56	20	15	12	41	18	14	44	38.5	54	8	Cabañas
cb	M	4	131	135	139	123	149	58.7	39	133	27.4	74	24.5	18	22	54.7	20	16.2	15.2	38.5	16	13	45	42.3	52.5	6	Acacias
cb	M	3	145	142	143	129	177	62	45.5	139	31.5	72	29.2	18	18	59.3	23.3	17	14	42.3	18	18	48	46	53	7	Cabañas
cb	M	5	135	128	126	120	155	56.5	43	123	29	65	21.5	17	16.5	55	22	16	14	39	17	14	44	41	52	7.5	Cabañas
cb	M	7	134.3	129.8	130.3	120	156	56.6	39	125	28	67	20	16.5	15	54.5	20	16.5	11.5	38	17.3	15	43.7	40.5	54.3	7	Cabañas
cb	M	5	136.5	138	139.4	122.9	164	59.8	45	132	29.7	76	36.5	18	19	53.4	20	19	16	34.4	16.7	14	43	46.5	52	8	Ranchito
cb	M	4	134	137	134	127	160	62.5	43.2	133.5	26.8	69.5	17	16.5	17	56	20	17	12	39	17	15	44	42	52	8	Cabañas
cb	M	6	137	141	140.5	123	161	58	48	135.5	27	71	21	17	17	58	21	16	13	42	19	15	46	43	56	8	Cabañas
cb	M	7	138	140	138	124	153	57	41	131	24	73	21	17	16	54	21	16	13.3	38	18	14.5	42	42.5	54	8	Cabañas
cb	M	8	147	142	144	134	169	52.2	46.2	135	28.5	72	23.5	19.8	24	59	22	16	14	43	16.5	15	46	46.5	57	8	Cabañas
cb	M	8	134	131	137	127	155	57	37	127	27	67	23	17.5	19	51	20	17	12	34	16	14	45	44	48	7.5	Bahía
cb	M	6	142	144	144	130	163	65	37	137	26	76	22	19	18	57	21	18	13	39	18	17	46	45	55	7.5	Bahía
cb	M	5	136.5	154.5	134	115	147	52	39	125	28.2	75	22.7	17.2	18	55	21	18	15.3	37	18.4	16.5	46	39.2	53	8	La_Paz
cb	M	15	127	133.2	131.5	117	148	56	41.5	126.6	31	68	34.5	24	18	53.5	19.5	22	13.5	31.5	16	15	42.3	41.3	55.3	8.5	B._Aires
cb	M	5	126.4	132	133.7	120.5	148	54	38	125	27	73.5	20.5	17	18	55.5	20	16	13	39.5	15.5	13	42	43	48.5	7	Zamuracos
cb	M	6	132.4	131.5	132.5	123	150	55	36	125	23	70	19	16.2	17	55	20.5	16	12.5	39	16.5	14	45	43.4	51	7	Zamuracos
cb	M	4	137	132	137	127.3	156	56	43	131.5	28.4	76	19.4	18.5	17	56	22	16.4	12.5	39.6	16.5	15	46	45	51.5	8	Zamuracos

cb	M	10	135.2	135.5	135.5	122	155	58	47.2	127	34	69.3	27	16.8	20.8	56	19	17.2	13.7	38.8	17	15.3	46.4	37	55	8.5	Arenosa
cb	M	20	131	136	137.6	122.7	159	57	42	128.5	32	71	22.5	18	17	57	22	17	13	40	17	15.5	45	44.6	53.5	8.5	Zamuracos
cb	M	9	132	131	133	116.5	159	57	41.4	127	32.5	64.5	25	18	17	54.2	20.2	16	15.2	38.2	16	12.5	44	40	50	7.5	La_Paz
cb	M	8	119.7	127.5	133.3	119	149	56	40	125.5	22	70	19	17	16	53	21	16.5	12	36.5	17	17	38	44	48	8	Delicias
cb	M	6	131.2	129.5	131	118	152	50	42.7	124	29.7	70	30	23.5	22.5	52	20.1	17.7	10	34.3	16.5	15	46.3	40.4	48.3	7	Belencito
cb	M	6	129	134	136	126	149	56	33	128	24	68	19.5	17.3	20	56	20	17	12	39	18	17	43	44	49	8	Bahía
cb	M	5	140	135.5	140	121	161	62	43	135	28	71	20.1	18.5	18	51.3	20.7	16.2	14.4	35.1	17.2	15	48	43.2	50.5	8	La_Paz
cb	M	7	135.5	131.2	128.3	120.3	155	58	38	126	27	71.5	21	18	17	57	20.5	17.5	14.5	39.5	16.7	15	45	42	53	8.5	Zamuracos
cb	M	2	120	126.5	131.3	117.3	137	53	35	122	24	68	16.5	15.5	15	51	20	15	12	36	16	16	43	39	48.5	9	Zamuracos
cb	M	6	128.7	136	135.5	121.5	153	56	45	131	30	71	23	17	16	54	20.3	15	13	39	17	16	46	46	52	7.5	Zamuracos
cb	M	5	135	134	132.2	116	152	55	46	130	27.3	71	23.3	17.5	15	51.8	19.5	15.1	13.7	36.7	17	15	42.4	41	50	8	La_Paz
cb	M	4	135	140	141	124	156	60.7	43	135	31	75	26	18	18	55.5	20.5	17	12.5	38.5	15.5	14	46.6	42	51.5	8	Libertad
cb	M	7	123	139.5	138.5	121.5	155	59.5	44.2	133	28.3	76	33.7	21.5	19	59	21.5	17	17.5	42	18	14	46	42	49.6	8	El_Secreto
cb	M	4	135	133	134	119	155	59	47.7	127	33	69	27.5	17.5	21	57	19	18	14	39	16.8	15	47.7	36.5	48	8.5	Libertad
cb	M	7	139	138.7	142	127.2	156	62	33	134.3	23	71	21	19.3	19	58	21	18	13	40	17	17	45	45	50	8	Bahía
cb	M	7	124	132	135	117.5	160	57	46	127	30.2	67.6	25.8	18	20	52.3	21.5	16	14	36.3	17	14	46	43	46.6	8.5	Libertad
cb	M	7	129.6	128.2	127	118	148	55.8	39.2	122	29.6	66.6	32.5	20	17	52.7	19.8	19.5	13.5	33.2	17.5	14.5	44.2	39.5	48	8	El_Secreto
cb	M	14	129.1	128.8	128.4	115.3	154	56	42	122.3	27.5	65	21	17	17	54	20	16	13	38	17	15	43	43	50.3	8	Chenchena
cb	M	18	128.3	129.2	129.8	117	152	57	36	122.6	27	65	19	17	18	55	21	16	13	39	16	15.5	44	41.5	46.7	8	Chenchena
cb	M	5	129.2	134.6	135.6	124.3	150	57	35	126.8	25.5	71	18	17	17	53	22	16	13	37	17.5	13	46	45	52.7	8	Chenchena
cb	M	8	133	136	136	118	159	59	40	130	27.5	71.5	20.6	17	17	55	21	16	13	39	17	16.5	44	43	52	8	Chenchena
cb	M	10	133.3	130.5	129	119.6	153	56	40	125.4	28	69	19	16	16	55	21	17	13	38	18	15.5	44	45	47	8	Chenchena
cb	M	9	142.7	142	142.7	129	163	61	40	137.3	28	71	18	18	18	59	23	19	14	40	19	16	46	43	53.2	7.5	Chenchena
cb	M	5	134	127	128.2	116.7	156	56	39	122.4	26	67	20	17	16	55	21	17	13	38	17	15	42	41	43.5	8	Chenchena
cb	M	7	135	128	132	118.8	149	56	38	122.6	28	66	18	18	16	56	20	16	13	40	17	15	43	41	45.7	7.5	Chenchena
cb	M	3	131	128.8	134	120.3	147	55	39	126	26	67	19	17	15	55	19	16	13	39	17	12	43	41	51	7.5	Chenchena
cb	M	3	130.3	130	131.3	120.6	151	57	37	126.3	25	68	20	17	16	53	20	16	12	37	16.4	14	42	42	52.7	7.5	Chenchena
cb	M	7	131.7	135.5	138.5	123.2	148	57	36	129.7	25.5	71	17	17	16	54	21	16	13	38	17	15	43	44	53.1	8	Chenchena

cb	M	3	138.2	138	136	126.5	155	57.3	42.4	133.4	27	73	22.4	17	16	54	20.2	14.2	13	39.8	17.3	15	46	39.3	52.8	8	Cabañas
cb	M	10	135.3	140	141.5	124.7	159	59	41	133.5	28	72	22	17	17	55	22	19	12	36	17	15	46	44	50.5	8	Delicias
cb	M	7	132.6	144	142.3	131	160	59	38	135.5	28	74	19	18	17	57	21	16	13	41	17.6	15	44	43	53.9	8	Delicias
cb	M	4	133	134.6	134	122.3	160	59.2	38.7	130	29	68	21	17	16.5	58.2	22	18	16	40.2	18.2	15	44	41	50	7.5	Cabañas
cb	M	8	122.5	142.5	142.3	129	163	60	41	136.7	30	77	22	17.5	16	55	21	17	12	38	17	15.5	45	46.5	53	7.5	Delicias
cb	M	9	133	144.2	144	130.6	163	62	37	137.4	26	76.5	22	17.5	17	57	21	16	12	41	18	14.5	46	45	55	7	Delicias
cb	M	5	131	138.4	142	130.6	161	61	39	133.6	30	70	20.5	17	16	56.5	22	16	12	40.5	16	16	45	45	56	7.5	Delicias
cb	M	6	139.5	138	146.5	131.6	159	57	40	135.4	25.5	77	20	17	16	57	21.5	18	12	39	19	14	47	46	52	7.5	Delicias
cb	M	7	125	130.3	132	116	152	57	34	125	27	65	21	16	15	56	19	18	12	38	18	15	42	42	49.8	7	Delicias
cb	M	5	133.5	126.5	129	129	148	56	37	120	27	66	19	16	15	58	19	18	11	40	17	15	44	43	51.7	7	Delicias
cb	M	7	131.4	142.5	142.3	129.3	160	59	39	135.7	30	77.8	22	17	16	56	21	17	12	39	18	15	45	45.5	50	8.5	Delicias
cb	M	9	139	141	140	125.3	161	61	39	132.7	27	71.5	19	17	16	58	22	17	13	41	17.5	16.5	45	45	52	7.5	Delicias
cb	M	5	127.4	138.4	141.5	126.5	154	58	40	134	28	74	19	16.5	16	54	21	16	12	38	17.5	15	44	44	49.5	7.5	Delicias
cb	M	10	136.5	145	143.5	130.7	162	60	38	141.3	28	76	21	17	16	56	22	16	12	40	19	15	44	46	52	7.5	Delicias
cb	M	4	130	131	132.7	120	141	54	33.7	123.9	26	69.8	16.6	17	16	51	19.5	15.7	11.1	35.3	16.2	15	44	40	44.3	7	Victoria
cb	M	6	132	135.5	138.4	123.5	152	58	41	130	24.5	73	20	16.5	17	55	21	16	13	39	17.5	17.5	45	44	46.4	8	Victoria
cb	M	3	133.5	141	139	125	155	58.7	45.3	133	29	71	19.4	18	18	56	20	16	12	40	18	13	47	41.6	51	8	Cabañas
cb	M	6	127	132	133	122.4	147	52	38	123.2	28.1	68	19.3	16	17	55	20	16	12	39	16	15	43	40.6	49.5	8	Victoria
cb	M	5	129	134	132.4	122	146	54	39.4	128	23	69.3	17.2	16.5	15.5	52.5	19.6	16	12.6	36.5	16.8	13.5	45	39.5	51	8	Victoria
cb	M	5	130	130.5	124.3	113.8	144	54	39	119.3	22.3	68.8	17.7	17	16.5	51	19	15	14	36	17	14	43	37.4	53	7.5	Victoria
cb	M	4	124	128	133	121	142	54	35	126	24.5	68	18.5	16	16	55	20.5	18	13	37	16.3	16	45	40	53	7.5	Victoria
cb	M	6	129	132	135	116.8	158	60	44.2	130	32.5	65.5	33.3	23	19.5	55	19.8	18	13.1	37	16.8	15.5	44	42.1	44.4	8	Nueva_Vid a
cb	M	5	131.5	134	132	124	161	62	46.2	130	35	70	36	20	18.5	54.5	20	17.4	12.5	37.1	17	16	45.5	44.8	50.7	8.2	Nueva_Vid a
cb	M	4	135	132	130	116	158	56.1	47.5	128	29.4	68.2	33.1	22	18	53	20	16.2	13.1	36.8	16	15.5	43.9	41	47.6	8.5	Nueva_Vid a
cb	M	4	132.4	133.4	136	130	158	60	37	130.3	27	67	25	17.5	20	57	20	16	13	41	17	15.5	46	48	48	8	Bahía
cb	M	4	136	136	133.2	126	161	60	44	133.2	27.5	68.5	20	16.5	16	55	20.5	16.5	12	38.5	16.6	16	44	41	51	8	Cabañas
cb	M	10	137	137	140	127	155	58	35	131	26	74	22	17	21	57	20	15	11	42	18	15	45	46	50	7	Bahía
cb	M	5	127	127	131	121	147	54	36	122	25	68	21	17	17	53	21	17	12	36	17	13	44	41	49	6.5	Bahía

cb	M	8	122	129	132	118	155	58	36	126	27	66	23	18	19	55	21	16	13	39	17	15	43	43	51	8	Bahía
cb	M	10	133	132	136	124	152	58	33	127	27	67	21	17	20	55	21	17	12	38	17	16	44	43	51	8	Bahía
cb	M	6	125	130	133	124	152	58	36	125	27	68	20	17	18	56	18	16	12	40	16	15	44	42	49	7.5	Bahía
cb	M	8	123	131	132	120	149	57	36	126	27	67	22	18	18	52	19	15.5	12	36.5	16	15	45	43	51	7.5	Bahía
cb	M	7	124	136	135	125	155	58	36	130.6	26	68	22	18	20	53	22	16	12	37	18	14	44	44	47	7	Bahía
cb	M	11	125.7	127.3	132	118	155	56	37	126.5	26.5	65	21	17	16	54	21	16	12	38	17	15	44	43	52	8	Bahía
cb	M	7	141	135	140	124	154	58	43.5	130	28	68	20.5	17.5	16	55	21	16	12	39	17	14	46	44	57	7.5	Cabañas
cb	M	8	140.7	138.5	139.5	127	163	59.5	50.3	131.2	35	74	37	19.5	20.5	57.2	21.2	19	13.4	38.2	17	17.5	48.4	45	52	7.1	Ranchito
cb	H	5	128	125	127.8	118	142	55	44	119	23	63	16	15	15	54	21	16	12	38	17	13	42	43	47.6	8	Zamuracos
cb	H	4.6	124.5	124	128.7	115	156	58.4	46	119	28	63	30	20.5	19.5	53	17.7	16	11	37	15.5	14	43.5	39.8	40.6	7	Belencito
cb	H	6	127.5	128.7	136.4	126	146	56	40	127.5	24	70	17.5	15	15	54	20.5	17	12	37	17	15	42	42	53	8	Zamuracos
cb	H	3	128	131	134	120	149	56	47.5	124.2	27.5	72	32	20.5	19.5	55	23	19	13	36	18	17.5	46.4	40	50	8	Belencito
cb	H	4	128.5	130	134	122	145	56	40	123	22.5	76	16.5	16	16	52	20	16	12	36	16	15	45	44	54	8.5	Zamuracos
cb	H	3	127	134	132	117	148	57	37	126	27	72	18	16	16	54	20	15	12	39	16	14.5	46	43	48	7	Zamuracos
cb	H	4	131.3	130.5	135.4	123.5	149	57	44	128	27	70	18.5	16	16	57	22	17	12	40	18	16	46	43	52.4	8	Zamuracos
cb	H	5	131.2	131.3	135.6	119	148	57	44	128	26.7	69	18.6	16	16	53	20	14	12	39	16	15	46	43	49	8	Zamuracos
cb	H	9	130	129.5	133	120	156	57.6	46.7	126	27	68	32	19	18.5	52	19.2	18	13	34	16	13	43	37	46.6	8.5	Belencito
cb	H	8	140	136.3	137.5	125.4	156	56.8	45.6	124	30	72	36	21	19.5	52.3	18.6	16.2	13	36.1	16	16.5	46.5	42.5	50	7.5	Belencito
cb	H	6	138	138.5	141	123.5	163	62.1	50.8	136.8	30.3	72	34.9	19	19.5	51.5	19.2	17.8	11.5	33.7	16.7	15.6	47	45	50	10.5	Ranchito
cb	H	8	135	136	134.8	126	146	56	41	125	23.5	71	16.5	16.5	16	56	22	17	13	39	16.3	16	45	44	48.9	8	Zamuracos
cb	H	6	143.5	138	134	124.9	163	62.5	50.3	130.2	31.2	73	35	23.5	22.5	57	19.8	17.2	12	39.8	17	16.5	44.5	45.7	48	8.5	Belencito
mu	M	6	118.4	122	122	111	136	53	32	120	26.5	64	19	17	15	52.5	20.2	15	13.7	37.5	16	19	39	34.5	46	11.5	Victoria
mu	M	14	128	131	132.3	113	149	58.7	42	128.1	27.5	63	24.3	17	17	56.8	20.7	17.3	15	39.5	17.5	21	41.5	37.6	44.3	10	Acacias
mu	M	4	119.5	122	122	106	138	54	32	119.5	23	67	19.5	14.5	16	52	20.3	16	14	36	16	19	39.5	35	49.5	10	Victoria
mu	M	4	119.5	122.4	122	112.5	141	54	35.5	117.5	23	68	20	14.5	14.5	51.3	19	16.3	13.8	35	15.7	19	39	35.5	44	9.5	Victoria
mu	M	4	117	118	120	108	132	51.8	33	115	20	65.8	17.5	14.5	13	54	20	17	11	37	16.3	19.5	38	33.7	43	9	Victoria
mu	M	10	114.5	117	117	107	138	53.2	33	116	19.5	61	17.5	15.5	15	50	20.1	17	12.5	33	16.3	19	35	33	50	10.5	Cabañas
mu	M	8	117	123	122	105	143	56.1	36.7	120	28	61	23.5	15	19	54.3	20.6	16.4	14	37.9	17	18	38.2	35.5	41.6	7.5	Acacias

mu	H	9	119	120.7	121.5	109.5	138	53	37	117.7	28.5	70	31.3	22	19	50	19.7	13.8	14.5	36.2	15.2	20	39.7	37.6	45.5	9.5	El_Secreto
mu	H	5	115	115.8	120.4	106	132	52	37	115	21	66	18.5	15	13	51.5	20	14.5	12	37	15.5	20	38	34	44.5	9	Victoria
mu	H	5	118.3	126.5	128.2	115	135	53	35.2	123.5	26.9	63.5	27	18.5	19	54	20	21	17	33	16.3	23.3	47	35.5	49.8	11	Mercedes
mu	H	10	120.5	132	136	117.5	164	60.5	44	135	30	70.3	35	18.5	18.5	54	20.7	20.5	13.5	33.5	18	20.5	42.3	40	48.5	11	Ranchito
mu	H	22	118	128	129.1	112	133	60	45	124	24	62.5	22	16	19	57	21.2	19.5	15.1	37.5	17.1	18	39.5	36	39.5	9	Acacias
mu	H	5	119	120	126	112	134	53.4	31	119	26.8	68	28.3	22.5	21.5	53.4	21.7	18.3	19.7	35.1	17	19	41.6	33.7	46.5	10	Mercedes
mu	H	5	125	133	131.8	116.5	151	59	35	127	28	65	20	16.5	16	57	23	19	16	38	19	20	42.5	40	43	10	Victoria
mu	H	8	125	125.2	122	107	140	56.5	36.8	118	25.6	65.8	21.6	17	19	54	21.5	16.5	13.2	37.5	16.7	19	41.2	36.2	45.5	8	Acacias

cb: horses; mu: mules; PT: heart girth circumference; LCO: body length; DDE: thoracic depth; DB: thoracic width; ALC: withers height; AHS: sternum height; ANESP: shoulders width; AP: chest width; PCA: forelimb cannon perimeter; LCÑ: forelimb cannon length (LCÑ); LCA: head length; ACA: head width; LCR: skull length; ACR: skull width; LC: face length; AC: face width; LORE: ear length; ORE: ear width; AL: loin height; ALGR: croup height; AGR: croup width; LGR: croup length; ANC: dock height; ACO: hock height.

Table S2. Loading values for Principal Components 1 (PC1) and 2 (PC2).

	**PC1**	**PC2**
LCO	0.4029	-0.2650
ALC	0.3238	0.0342
ALGR	0.3528	0.1966
ANC	0.3840	0.0094
PT	0.4296	-0.2689
DDE	0.0836	0.1072
DB	0.2071	0.1822
AL	0.2242	0.1246
ANESP	0.0907	0.1934
ALES	0.1667	0.1041
ANT	0.0771	0.5706
PCA	0.0594	0.2807
LCÑ	0.0395	0.2605
LCA	0.0374	0.0614
ACA	-0.0011	0.0998
LCR	-0.0079	0.1814
ACR	-0.0419	0.2319
LC	0.0453	-0.1199
AC	0.0088	0.0413
LORE	-0.1425	0.2225
LGR	0.1636	0.2012
AGR	0.2273	-0.0086
ACO	0.1472	-0.1662
ORE	-0.0577	0.0526

PT: heart girth circumference; LCO: body length; DDE: thoracic depth; DB: thoracic width; ALC: withers height; AHS: sternum height; ANESP: shoulders width; AP: chest width; PCA: forelimb cannon perimeter; LCÑ: forelimb cannon length (LCÑ); LCA: head length; ACA: head width; LCR: skull length; ACR: skull width; LC: face length; AC: face width; LORE: ear length; ORE: ear width; AL: loin height; ALGR: croup height; AGR: croup width; LGR: croup length; ANC: dock height; ACO: hock height.

Figure S1.

	Sex	PC1	PC2
cb	M	-5.160	-1.374
cb	M	5.370	11.023
cb	M	2.148	7.120
cb	M	-15.418	4.000
cb	M	-8.674	-2.805
cb	M	7.157	-3.388
cb	M	-9.786	-1.261
cb	M	-17.962	-6.690
cb	M	-13.583	-1.715
cb	M	6.556	11.877
cb	M	6.959	-1.527
cb	M	13.475	8.615
cb	M	18.624	5.895
cb	M	4.008	8.563
cb	M	7.491	-1.096
cb	M	-7.380	-2.319
cb	M	-9.772	-2.303
cb	M	-9.478	-5.274
cb	M	3.143	11.301
cb	M	-6.036	-0.179
cb	M	-8.833	8.549
cb	M	2.897	13.017

cb	M	0.334	9.330
cb	M	0.348	0.049
cb	M	-5.137	10.578
cb	M	4.985	13.280
cb	M	-1.093	9.215
cb	M	-15.683	4.831
cb	M	22.139	9.279
cb	M	-11.050	6.823
cb	M	19.518	13.678
cb	M	32.527	11.031
cb	M	6.600	7.375
cb	M	-12.262	-2.881
cb	M	1.267	1.186
cb	M	4.163	6.386
cb	M	-11.066	-8.493
cb	M	-10.151	-3.178
cb	M	3.591	-3.407
cb	M	-3.048	-4.312
cb	M	-22.249	3.846
cb	M	-15.528	-3.857
cb	M	-31.516	-7.733
cb	M	15.045	-1.356
cb	M	-0.255	0.966
cb	M	20.416	1.145
cb	M	-1.168	-0.560
cb	M	24.727	2.448
cb	M	5.380	-4.542
cb	M	7.478	10.406

cb	M	5.880	10.150
cb	M	20.486	-5.670
cb	M	-4.525	-5.972
cb	M	17.179	-2.752
cb	M	18.865	-2.875
cb	M	9.506	-5.386
cb	M	4.082	-4.498
cb	M	4.791	3.397
cb	M	32.691	-0.489
cb	M	-3.156	-6.826
cb	M	-1.138	-8.672
cb	M	16.996	6.998
cb	M	10.277	-6.685
cb	M	16.977	-3.388
cb	M	9.257	-4.912
cb	M	31.154	-3.972
cb	M	4.024	-2.631
cb	M	24.220	-2.555
cb	M	4.029	0.112
cb	M	-4.653	9.872
cb	M	-5.147	-2.230
cb	M	-2.180	-6.912
cb	M	10.761	-3.476
cb	M	6 835	2.601
cb	M	8.253	-1.259
cb	M	0.211	-2.256
cb	M	-11.437	-2.598
cb	M	-3.291	5.098

cb	M	-1.575	-2.861
cb	M	13.721	-2.685
cb	M	0.115	-5.765
cb	M	-20.952	-3.173
cb	M	4.511	0.548
cb	M	0.242	-2.446
cb	M	13.934	3.252
cb	M	6.851	12.580
cb	M	3.536	4.359
cb	M	12.843	-2.636
cb	M	0.409	3.318
cb	M	-9.737	5.041
cb	M	-7.391	-5.367
cb	M	-9.254	-5.314
cb	M	0.538	-4.679
cb	M	5.275	-4.320
cb	M	-2.877	-5.914
cb	M	21.846	-4.864
cb	M	-6.783	-7.449
cb	M	-6.505	-6.416
cb	M	-5.460	-5.728
cb	M	-4.561	-6.563
cb	M	1.454	-4.896
cb	M	10.819	-3.553
cb	M	13.667	-1.001
cb	M	17.230	-3.544
cb	M	5.044	-3.931
cb	M	15.615	1.637

cb	M	21.216	-2.475
cb	M	15.221	-3.336
cb	M	20.837	-3.359
cb	M	-9.325	-4.856
cb	M	-4.454	-8.163
cb	M	16.846	-0.135
cb	M	15.588	-5.919
cb	M	8.001	-0.919
cb	M	22.467	-2.962
cb	M	-11.325	-4.500
cb	M	4.116	-1.131
cb	M	11.587	-1.493
cb	M	-7.004	-3.893
cb	M	-4.963	-5.332
cb	M	-15.311	-8.080
cb	M	-12.002	-2.382
cb	M	1.676	9.007
cb	M	9.595	8.144
cb	M	2.064	4.302
cb	M	8.776	-0.818
cb	M	9.923	-6.023
cb	M	11.147	-3.301
cb	M	-10.132	-3.803
cb	M	-7.098	-1.557
cb	M	0.337	-3.706
cb	M	-4.629	-3.542
cb	M	-7.721	-1.027
cb	M	0.844	-0.305

cb	M	-6.280	-5.108
cb	M	11.545	-5.453
cb	M	21.779	10.115
mu	M	-32.976	-2.628
mu	M	-10.574	2.741
mu	M	-32.820	-4.135
mu	M	-29.299	-3.753
mu	M	-40.639	-5.292
mu	M	-41.229	-7.598
mu	M	-31.365	0.649
mu	H	-29.407	7.819
mu	H	-41.613	-3.651
mu	H	-24.820	10.527
mu	H	1.708	11.261
mu	H	-25.730	7.723
mu	H	-30.790	10.123
mu	H	-10.247	0.270
mu	H	-26.737	-1.108
cb	H	-15.365	-7.568
cb	H	-10.829	3.207
cb	H	-3.780	-4.596
cb	H	-2.200	9.869
cb	H	-4.948	-5.142
cb	H	-6.172	-4.012
cb	H	0.689	-2.844
cb	H	-1.910	-3.239
cb	H	-1.029	4.609
cb	H	11.529	6.891

cb	H	19.685	8.570
cb	H	1.176	-4.933
cb	H	18.810	6.846

cb: horses; mu: mules
